# Binary similarity measures for fingerprint analysis of qualitative metabolomic profiles

**DOI:** 10.1007/s11306-018-1327-y

**Published:** 2018-01-31

**Authors:** Anita Rácz, Filip Andrić, Dávid Bajusz, Károly Héberger

**Affiliations:** 1 Plasma Chemistry Research Group, Research Centre for Natural Sciences, Hungarian Academy of Sciences, Magyar tudósok krt. 2, Budapest, 1117 Hungary; 2 Department of Analytical Chemistry, University of Belgrade - Faculty of Chemistry, Studentski trg. 12-16, 11000 Belgrade, Serbia; 3 Medicinal Chemistry Research Group, Research Centre for Natural Sciences, Hungarian Academy of Sciences, Magyar tudósok krt. 2, Budapest, 1117 Hungary

**Keywords:** Plant metabolomics, Qualitative metabolomic data, Binary similarity measures, Fingerprint analysis

## Abstract

**Introduction:**

Contemporary metabolomic fingerprinting is based on multiple spectrometric and chromatographic signals, used either alone or combined with structural and chemical information of metabolic markers at the qualitative and semiquantitative level. However, signal shifting, convolution, and matrix effects may compromise metabolomic patterns. Recent increase in the use of qualitative metabolomic data, described by the presence (1) or absence (0) of particular metabolites, demonstrates great potential in the field of metabolomic profiling and fingerprint analysis.

**Objectives:**

The aim of this study is a comprehensive evaluation of binary similarity measures for the elucidation of patterns among samples of different botanical origin and various metabolomic profiles.

**Methods:**

Nine qualitative metabolomic data sets covering a wide range of natural products and metabolomic profiles were applied to assess 44 binary similarity measures for the fingerprinting of plant extracts and natural products. The measures were analyzed by the novel sum of ranking differences method (SRD), searching for the most promising candidates.

**Results:**

Baroni-Urbani–Buser (BUB) and Hawkins–Dotson (HD) similarity coefficients were selected as the best measures by SRD and analysis of variance (ANOVA), while Dice (Di1), Yule, Russel-Rao, and Consonni-Todeschini 3 ranked the worst. ANOVA revealed that concordantly and intermediately symmetric similarity coefficients are better candidates for metabolomic fingerprinting than the asymmetric and correlation based ones. The fingerprint analysis based on the BUB and HD coefficients and qualitative metabolomic data performed equally well as the quantitative metabolomic profile analysis.

**Conclusion:**

Fingerprint analysis based on the qualitative metabolomic profiles and binary similarity measures proved to be a reliable way in finding the same/similar patterns in metabolomic data as that extracted from quantitative data.

**Electronic supplementary material:**

The online version of this article (10.1007/s11306-018-1327-y) contains supplementary material, which is available to authorized users.

## Introduction

Contemporary metabolomic fingerprinting is relatively fast, providing extensive information about relationships among samples, chemical and functional diversity of living organisms (Ivanišević et al. 2011), and has important roles in: (a) discovery of novel bioactive compounds, (b) chemotaxonomic evaluation of organisms (Christensen et al. 1999; dos Santos et al. 2017; Farag et al. 2012a, 2013b; Ivanišević et al. 2011; Jing et al. 2015), (c) quality control of herbal preparations and natural products (Farag et al. 2013a; Farag and Wessjohann 2012), (d) elucidating causative relations between exogenous factors and metabolic changes in organisms (Allwood et al. 2008; Krstic et al. 2016; Shulaev et al. 2008; Xie et al. 2014), and (e) tracking metabolome differences influenced by geographic origin (Farag et al. 2012b; Krstic et al. 2016).

In the simplest form metabolomic fingerprinting is based on pure analytical signals excluding any direct chemical information (Anđelković et al. 2017). Nevertheless, multivariate methods, such as principal component analysis (PCA), or hierarchical cluster analysis (HCA) can further identify the signals originating from a single metabolite or a group of metabolites responsible for sample separations (Farag et al. 2013a, 2012, 2012a; Ivanišević et al. 2011; Porzel et al. 2014). Another, completely different approach starts from the identification of signal sections such as well separated chromatographic peaks, careful analysis and assignments of metabolites to each of them (after spectral library and literature search, and/or confirmation with standard compounds) (Farag et al. 2013a; Jing et al. 2015; Kicel et al. 2016), and then subjecting absolute peak areas or their ratios to PCA or HCA (Jing et al. 2015; Kicel et al. 2016). The main drawback of signal-based comparison is the lack of comprehensive chemical information, which can be obtained only by quantitative analysis. However, quantification of all present compounds in plant extracts is almost an impossible task. At best, only few prominent markers are determined (Farag and Wessjohann 2012).

On the other hand, qualitative metabolomic data encoded only by the presence or absence of particular metabolites is on the rise (Arsenijević et al. 2016; Cardarelli et al. 2017; Dimkić et al. 2016; Kicel et al. 2016; Liu et al. 2017; Mišić et al. 2015; Mkrtchyan 2014; Xu et al. 2011). Although such approaches inevitably suffer from some information loss, their usage has several advantages. First, the use of complex instrumentation necessary to accurately resolve convoluted signals can be avoided. Second, the tedious quantification step is avoided. Finally, the analysis time and costs are significantly reduced.

Such types of data where the presence of a particular metabolite is denoted by 1 and the absence by 0 are called binary metabolomic data. Dealing with binary metabolomic profiles is not a novelty, and several statistical approaches have been already meticulously studied by Frisvad and coworkers few decades ago, mostly related to HCA, correspondence analysis (CA), and principal coordinate analysis (PCO) applied to fungi taxonomy (Banke et al. 1997; Christensen et al. 1999; Frisvad 1992, 1994; Larsen and Frisvad 1995). The authors confirmed an improved clustering and separation of taxa by the combination of quantitative and qualitative binary data (Frisvad 1994), or even just by binary metabolomic data (Larsen and Frisvad 1995). However, dealing with binary metabolomic data requires the use of various similarity metrics, which will be explained in the following section.

### Similarity measures for binary data

Similarity metrics are used to compare binary and continuous data vectors across the whole spectrum of scientific fields, although it is worth to note that the fields of taxonomy and ecology have been particularly active with regard to proposing novel similarity metrics to classify various sorts of species and their associations (Dice 1945; Faith et al. 1987; Rogers and Tanimoto 1960; Russell and Rao 1940). Similarly, many metrics have been contributed by statisticians (Peirce 1884; Sokal and Michener 1958; Yule 1900). To our knowledge, the most comprehensive collection and comparison of similarity metrics was published by Todeschini et al. (2012). They have compiled a list of 51 similarity metrics, out of which seven have been shown to perfectly correlate with others.

For binary data, similarity metrics are calculated from a contingency table that summarizes the occurrences of the possible permutations of a feature (here, metabolite) between two samples: 1–1 (metabolite present in both samples), 1–0 (metabolite present in the first sample and absent in the second), 0–1 (metabolite absent in the first sample but present in the second), and 0–0 (metabolite absent from both samples). Frequencies of these events for all metabolites between two samples are here denoted as *a, b, c* and *d* respectively, and the total number of metabolites is *p*, which by definition equals *a* + *b* + *c* + *d* (see Online Resource 1, Table OR1). With these parameters, various similarity metrics can be calculated, as exemplified here:

1 $$SM=\frac{{a+d}}{p}$$

2 $$JT=\frac{a}{{a+b+c}}$$

3 $$CT5=\frac{{\ln (1+ad) - \ln (1+bc)}}{{\ln \left( {1+{p^2}/4} \right)}}$$

Here, *SM* is the simplest similarity coefficient (called *simple matching*, or *Sokal–Michener*), *JT* corresponds to the *Jaccard–Tanimoto* coefficient, which is the most popular choice of cheminformaticians for molecular similarity calculations (Bajusz et al. 2015), and *CT5* is a novel similarity measure introduced in (Consonni and Todeschini 2012).

The values of similarity usually range from 0 to 1 (as for *SM* and *JT* from the above examples), but that is not always the case, for example the *CT5* metric (along with a number of correlation-based similarity metrics) ranges from − 1 to + 1. Such metrics are rescaled to the range [0,1], based on the simple transformation below:

4 $$s^{\prime}=\frac{{s+\alpha }}{\beta }$$

where *α* and *β* are the scaling parameters compiled by Todeschini et al. (2012). The same paper also covers in great detail categorizations of similarity metrics according to concordance symmetry and metricity. The former differentiates the metrics whether they consider the frequencies of *d* equally to the frequencies of *a* (*symmetric, S*), underweighted with respect to *a* (*intermediate, I*), or not consider it at all (*asymmetric, A*). Correlation-based metrics that are transformed to the [0,1] range are labeled with *Q*. Metricity differentiates whether a similarity measure can be transformed into a metric distance (i.e. one that complies with the non-negativity, identity of indiscernibles, symmetry and triangle inequality, denoted with *M*) or not (*N*).

### Aims

Taking into account a great number of binary similarity metrics that can be used to group, cluster or classify samples and metabolites, and their various sensitivities to binary metabolome structure, the inevitable question is which ones are the best, and which ones should be avoided?

Using a consensus-based non-parametric comparison, our aims were to: (i) identify the most appropriate and the least suitable binary similarity coefficients, (ii) establish whether qualitative (binary) metabolomic information can reveal the same or highly similar patterns among samples and metabolites as contemporarily used metabolomic fingerprinting based on quantitative information. As we will see later, the approach based on binary qualitative metabolomic data resulted in very similar patterns as the ones obtained by quantitate metabolomic approach when using unsupervised pattern recognition techniques, i.e. hierarchical cluster analysis.

## Methodology

### Metabolomic data collection

Nine different metabolomic datasets were selected for the comparison of similarity metrics. Special care was taken regarding the dataset size (number of samples and metabolites), types of metabolites, analytical methods, and application field. Every dataset is represented by a binary table with samples arranged in rows and metabolites arranged in columns. The presence and absence of metabolites were indicated by 1 and 0, respectively. Short descriptions of the datasets are summarized in Table 1. The Dimkić et al. dataset was split into three parts based on the type of the measured compounds (phenolic acids and esters, flavonoids, glycerides and glycosides). Complete data sets can be found in Online Resource 2.

**Table 1:** Case studies (summary)

Dataset	Reference	Analysed material	Metabolites	No. of metabolites	No. of samples	Analytical method
1	Arsenijević et al.	Hungarian thyme	Polyphenolic compounds	12	8	HPLC-DAD
2	Cardarelli et al.	Aloe species		16	18	UHPLC-QTOF
3	Dimkić et al.	Plant resins and propolis	Carboxylic acids, phenolic acids and esters	26	17	UHPLC–MS/MS Orbitrap
4	Dimkić et al.	Plant resins and propolis	Flavonoids	26	17	UHPLC–MS/MS Orbitrap
5	Dimkić et al.	Plant resins and propolis	Glycerides and glycosides	11	17	UHPLC–MS/MS Orbitrap
6	Kicel et al.	*Cotoneaster Medik*. species	Polyphenols	34	12	UHPLC-PDA-ESI-QTOF-MS
7	Mišić et al.	*Nepeta* species	Phenolic acids and their derivatives	37	12	UHPLC-LTQ/orbitrap-MS
8	Mrktchyan et al.	Coprinoid mushrooms (*Coprinellus*)	Fatty acids	5	17	GC (FID)
9	Xu et al.	Grapes, grape-derived products	Polyphenols	53	29	HPLC-MS (DAD, MSD trap, ESI)

### Selection of similarity measures for qualitative metabolomic data

In total, 44 similarity measures have been selected, with 13 concordantly symmetric, 17 asymmetric, 2 of intermediate symmetry and 12 correlation-based ones. Half of them (*n* = 22) were metric and the second half non-metric. The same notation as in the work of Todeschini et al. (2012) was used. Definitions, labels, and names of similarity metrics are given in the Online Resource 1, Table OR2.

### Sum of ranking differences

Sum of ranking differences (SRD) is a novel, general method for the ranking and comparison of models, metrics, techniques (Héberger 2010; Kollár-Hunek and Héberger 2013). It is based on the following steps: (1) start with an input matrix, with the variables (similarity metrics) in the columns and the samples in the rows, (2) add a reference column, that can be either a gold standard, or a consensus of the variables (row-wise average, maximum or minimum, depending on the dataset), (3) rank transform each column (including the reference) by increasing magnitude, (4) calculate the differences between the ranks of each variable and the reference for each sample, (5) sum up the absolute differences for each variable. The latter are called SRD (sum of ranking differences) values and they represent the closeness to (or consistency with) the ranking pattern of the reference method (the smaller the better). For better comparability, the normalized (scaled) versions of SRD values are given and plotted, along with the distribution of SRD values for randomized rank numbers. The procedure is explained in animated plots in the recent work of Bajusz et al. (2015). SRD is further validated with bootstrap (repeated and randomized) cross-validation.

SRD is developed as an MS Excel macro, and is available for download at: http://aki.ttk.mta.hu/srd.

### Other statistical methods

Analysis of variance (ANOVA) was used for the comparison of the similarity metrics based on the SRD values. This method is based on the pairwise comparison of the average values of the different groups of samples. STATISTICA 13 (Dell Inc., Tulsa, OK, USA) was used for the analysis. Different factors such as classes and metricity were compared separately.

## Results and discussion

### Consensus-based comparison of similarity measures

**Fig. 1:**
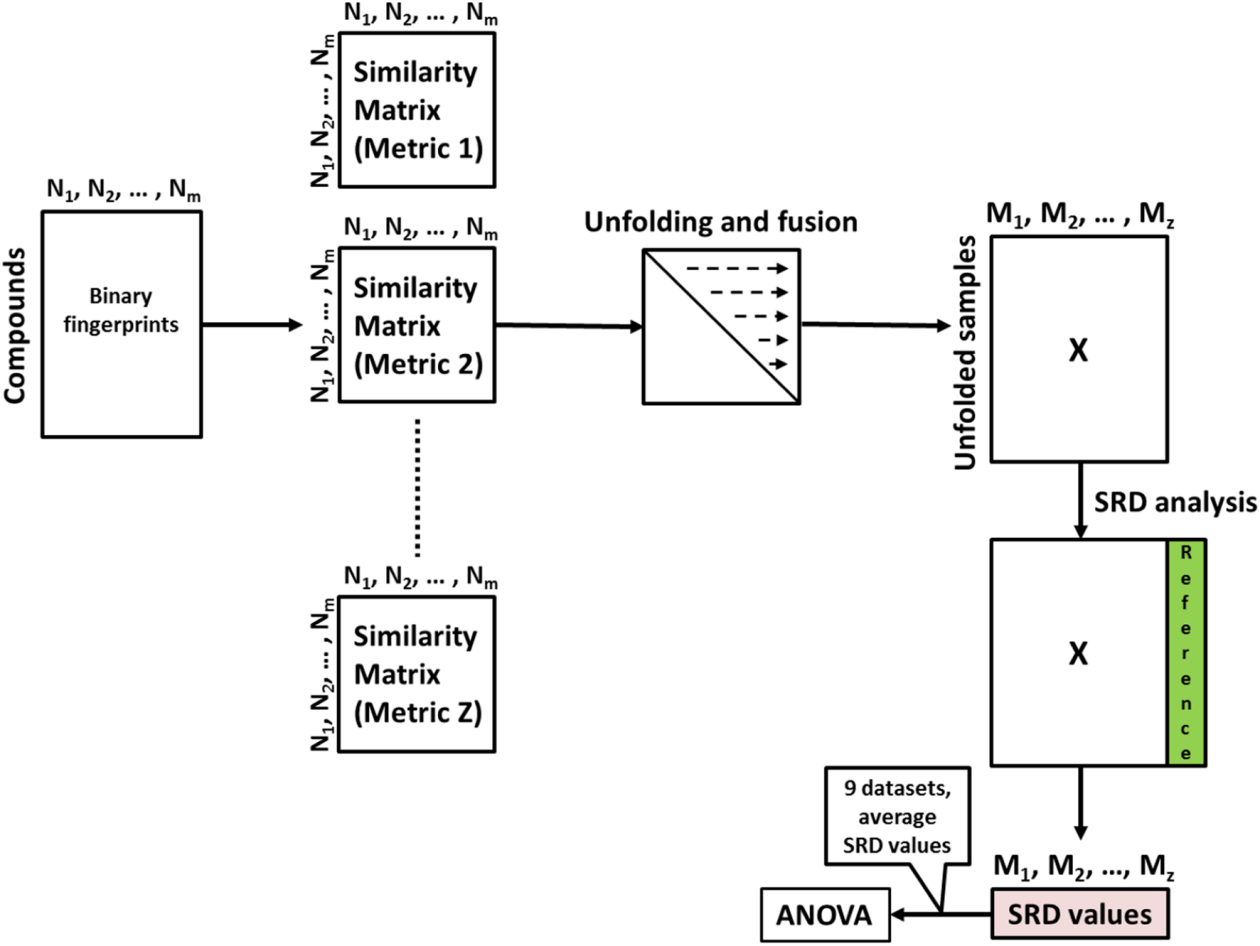
Workflow of the comparison procedure. Binary fingerprints encode the presence or absence of a compound in a sample (N_1_ to N_m_). For each similarity metric (M_1_ to M_z_) a full similarity matrix is calculated and then “unfolded” (or “flattened”) to a single vector. The average and normalized SRD values of more than 50 bootstrap analyses per datasets were used for ANOVA. (Color figure online)

Starting from binary fingerprints, the workflow of the calculation and comparison procedure is depicted in Fig. 1.

**Fig. 2:**
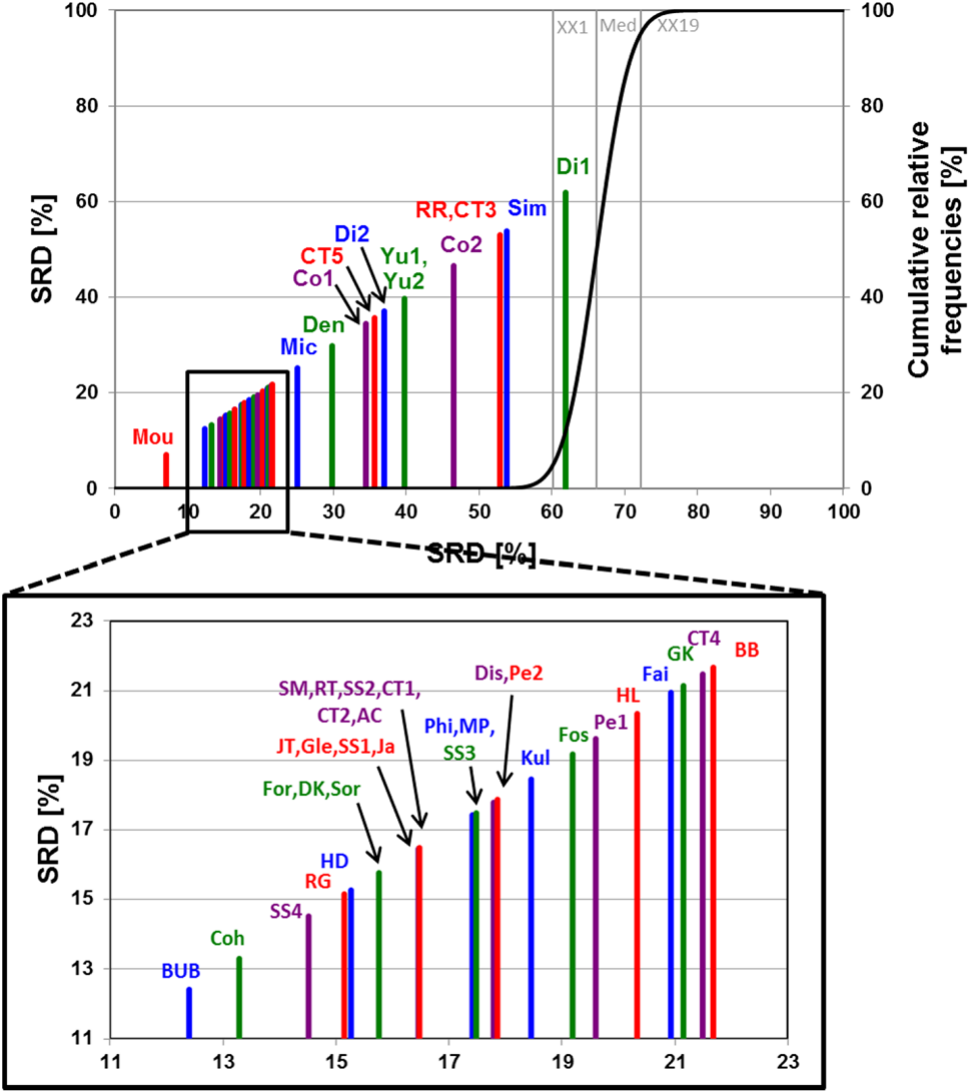
One example of the SRD results (Dataset 3). Normalized SRD values (percentages) are plotted on the X and left Y axes. The cumulative relative frequencies of SRD values in the randomization test (%) are plotted on the right Y axis. (The original plot was magnified for better visualization). (Color figure online)

For each similarity metric (44), a full similarity matrix was calculated and “unfolded” to a single vector (Haws et al. 2012). These vectors were compiled in a final **X** matrix (with the similarity metrics in the columns and the unfolded similarity matrix elements in the rows) for the SRD analysis with the row-wise average as the reference column, and bootstrap cross-validation (more than 50 rounds of SRD for each dataset). One example of the SRD evaluations can be seen in Fig. 2.

**Fig. 3:**
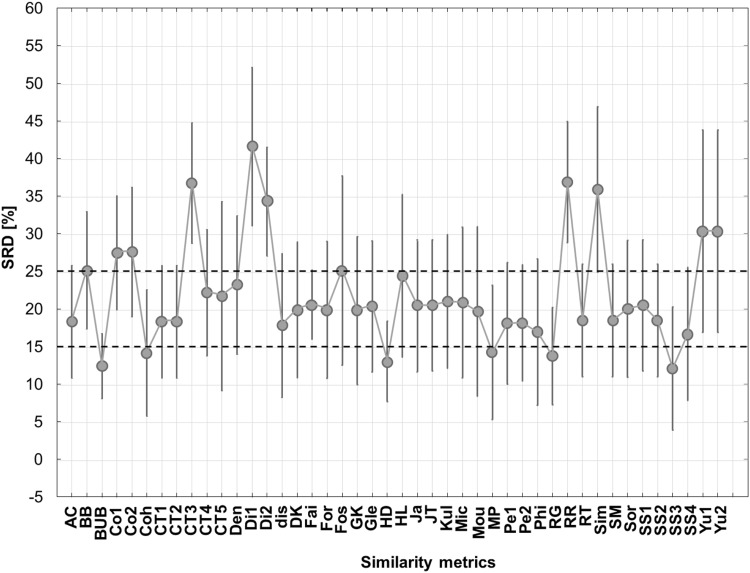
ANOVA decomposition of similarity metrics as factor. Dashed lines symbolize the limit of the best/consistent (lower part), worst (upper part) and medium groups of similarity metrics based on SRD values. 95% confidence limits are plotted with vertical bars. (Color figure online)

Mean SRD values were calculated and normalized for the appropriate comparison between the nine datasets with ANOVA. First, the similarity metrics were used as the factor for the analysis: in this case the similarity metrics were significantly different (α = 0.05, see the averages and the 95% confidence intervals in Fig. 3). The similarity metrics can be split to three groups based on this plot: those having smaller SRD values than 15 can be considered the most consistent based on the 9 datasets. These are BUB (Baroni-Urban–Buser) and HD (Hawkins–Dotson), followed by Coh (Cohen), MP (Maxwell–Pilliner), RG (Rogot–Goldberg) and SS3 (Sokal–Sneath). Metrics between SRD values of 15 and 25 are in the medium group, while the weakest ones have SRD values greater than 25.

**Fig. 4:**
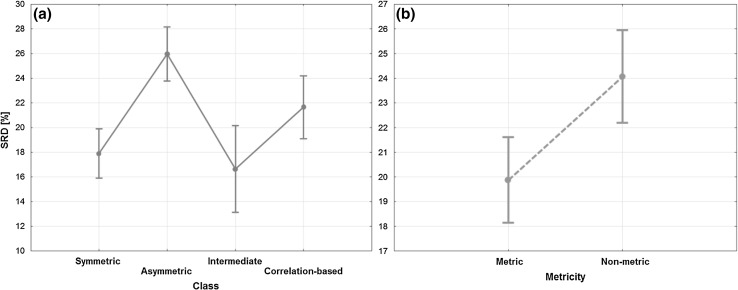
ANOVA decomposition of factors: classes (**a**) and metricity (**b**). Vertical lines denote the 95% confidence intervals around the average values. (For **b**, notice the lack of overlap between the confidence intervals). (Color figure online)

Similarity metrics can be grouped into four different classes: symmetric, asymmetric, intermediate and correlation-based. ANOVA was also carried out with these classes as the factor for the analysis, and the differences were, again, statistically significant. As seen in Fig. 4a, the best ones were the symmetric (and intermediate) metrics, while the weakest one was the asymmetric group. Based on the Tukey and Bonferroni post-hoc tests, the asymmetric class clearly differs from the others and the other three classes overlap.

The superiority of symmetric (and intermediate) coefficients contrasts with cheminformatics, where usually asymmetric measures are preferred, although this is mostly explained by the usually greater sparsity of molecular fingerprints (Todeschini et al. 2012).

Metric and non-metric groups were used as the factor in ANOVA, as well. The two groups were significantly different (with the metric group being much better than the non-metric) and the results can be seen in Fig. 4b.

### Comparison of qualitative and quantitative metabolomic profiling

**Fig. 5:**
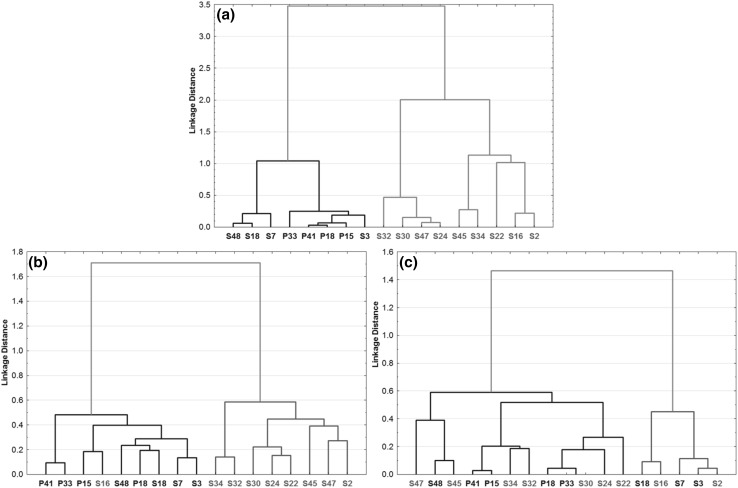
Comparison of cluster analysis trees (linkage rule: Ward’s method). **a** Reference (quantitative results). **b** Binary fingerprints with the BUB distance metric. **c** Binary fingerprints with Di1 distance metric. The two largest clusters (indicated with red and blue) were compared. It is clearly seen that the number of misclassifications (as compared to the reference) is one for the BUB, and 10 for the Di1 measure. (Color figure online)

The findings were tested on the Dimkić et al. dataset, because here the quantitative concentration data can be used as a reference set. The best and worst cases of binary similarity metrics were chosen and compared with the reference one. Cluster analysis was applied to the BUB (best) and Di1 (worst) distance matrices with Ward’s method as the linkage rule. In the same way we performed cluster analysis to the standardized and transformed (1 – |Pearson coeff.|) quantitative data as well. The comparison to the reference clustering (Fig. 5a) can be seen in Fig. 5. The use of the BUB distance metric for the distance matrix gave a 94.5% correct classification rate (CCR%) compared to the clusters of the reference. In this sense, the Di1 metric gave only CCR% = 45.5%, which is completely random. Thus with the use of the BUB metric the results are almost the same as in the case of continuous, quantitative data.

### Comparison with earlier literature findings

A recent work that shows some similarity to our approach was published in 2017 and deals with the classification of plants based on metabolite content (Liu et al. 2017). The basic assumption of the authors was that the similarity in metabolite content is applicable to assess the phylogenic similarity of higher plants. A particular difficulty of the applied taxonomic approach is the incompleteness of the metabolomics data. Nonetheless, the authors could successfully classify 216 plants based on their known (incomplete) metabolite content. While they have not used binary similarity coefficients, the plants have been represented as binary vectors, implying relations with structurally similar metabolite groups, and classified using hierarchical clustering with Ward’s method.

Metabolite identification is routinely done using spectral similarity measures; a spectral alignment algorithm establishes a “similarity score” between individual spectra. However, these are non-binary similarity metrics, even if some structural fragment is binarily encoded (presence/absence) (Allard et al. 2017).

In the work of O’Hagan and Kell, two binary similarity metrics (Tanimoto and Tversky) were applied for a maximum common substructure-based analysis of drugs and human metabolites. The molecular fingerprint (that was used to encode the molecular structures) had a dramatic effect on the apparent similarities observed. By contrast, the maximal common substructure (MCS) approach provided a means of determining similarities that is largely independent of the fingerprint type (O’Hagan and Kell 2017).

Recently, an efficient method was suggested to find both frequent closed itemsets and biclusters in high-dimensional binary data (Király et al. 2014). While the original publication appeared outside of the metabolomics field, the described method should be readily available for binary metabolomics data as well.

In a 2003 article by Heymans and Singh, binary relations between enzymes were established by comparing metabolic pathways in different genomes (Heymans and Singh 2003). The authors have applied a graph-based approach with several non-binary similarity measures calculated from the structural relationship between the enzymes (represented as graph nodes). The obtained phylogenetic trees closely matched existing phylogenies and revealed interesting relationships among organisms.

## Conclusion

Based on qualitative binary fingerprints, 44 similarity measures were compared on metabolomics datasets. SRD and ANOVA showed that the most consistent similarity measures are the Baroni-Urbani–Buser (BUB) and Hawkins–Dotson (HD) metrics, being fit for the replacement of quantitative data in cluster analysis tasks as well. Concordantly, intermediate and symmetric similarity coefficients are good candidates for metabolomic fingerprinting in general. The metric group of similarity measures was significantly better than the non-metric.

Similarity/distance metrics usually lead to different results and conclusions in cluster analysis, thus finding and using the most consistent metrics is an important part of this type of evaluations. The qualitative metabolomic profiles and binary similarity measures proved to be a reliable way in finding patterns in metabolomic data. Comparison with the cluster analysis based on quantitative profiles has corroborated our earlier conclusions.

## Electronic supplementary material

Below is the link to the electronic supplementary material.

Supplementary material 1 (DOCX 75 KB)

Supplementary material 2 (XLSX 44 KB)
