# Supplementary material for: Binary similarity measures for fingerprint analysis of qualitative metabolomic profiles
Source: Metabolomics. 2018 Jan 31;14(3):29. doi: 10.1007/s11306-018-1327-y (PMC5846857; doi:10.1007/s11306-018-1327-y)
Supplement: Supplementary file 1 — Supplementary material 1 (DOCX 75 KB) [file 11306_2018_1327_MOESM1_ESM.docx]

**SUPPLEMENTARY MATERIAL**

**Online resource 3**

**Binary similarity measures for fingerprint analysis of**

**qualitative metabolomic profiles**

Anita Rácz^1^, Filip Andrić^2,*^, Dávid Bajusz^3^, Károly Héberger^1^

^1^ *Plasma Chemistry Research Group, Research Centre for Natural Sciences,*

*Hungarian Academy of Sciences, H-1117 Budapest, Magyar tudósok krt. 2, Hungary;*

^2^ *Faculty of Chemistry, University of Belgrade, Studentski trg 12-16, 11000 Belgrade, Serbia*

^3^ *Medicinal Chemistry Research Group, Research Centre for Natural Sciences, Hungarian Academy of Sciences, H-1117 Budapest, Magyar tudósok krt. 2, Hungary*

*To whom correspondence should be addressed:

Dr. Filip Andrić

Assistant professor

Department of Analytical Chemistry

Faculty of Chemistry, University of Belgrade

E-mail: [andric@chem.bg.ac.rs](mailto:andric@chem.bg.ac.rs)

Phone: +381 11 3336 755

**CONTENTS**

**Table OR1** Contingency table for a pair of samples p. 2

**Table OR2** List of the binary similarity coefficients, their definitions, concordance symmetry and metric properties p. 3-6

**Table OR1** Contingency table for a pair of samples, containing the frequencies of common present (*a*), common absent (*d*), and exclusive metabolites for Sample 1 (*b*) and Sample 2 (*c*).

| *p = a + b + c + d* | | Sample 2 | |
| --- | --- | --- | --- |
|  |  | 1 (metabolite present) | 0 (metabolite absent) |
| Sample 1 | 1 (metabolite present) | *a* | *b* |
|  | 0 (metabolite absent) | *c* | *d* |

**Table OR2.** List of the binary similarity coefficients, their definitions, concordance symmetry and metric properties. The notations and classification of similarity measures were kept the same as in the work of Todeschini *et al*. 2014

| **No** | **Label** | **Name** | **Equation** | **Scaling parameters** | | **Concordance symmetry** | **Metricity** |
| --- | --- | --- | --- | --- | --- | --- | --- |
|  |  |  |  | α | β |  |  |
| 1 | SM | Simple matching,  Sokal-Michner | $s_{SM}=\frac{a+d}{p}$ | 0 | 1 | S | M |
| 2 | RT | Rogers-Tanimoto | $s_{RT}=\frac{a+d}{p+b+c}$ | 0 | 1 | S | M |
| 3 | JT | Jaccard-Tanimoto | $s_{JT}=\frac{a}{a+b+c}$ | 0 | 1 | A | M |
| 4 | Gle | Gleason | $s_{Gle}=\frac{2a}{2a+b+c}$ | 0 | 1 | A | N |
| 5 | RR | Russel-Rao | $s_{RR}=\frac{a}{p}$ | 0 | 1 | A | M |
| 6 | For | Forbes | $s_{For}=\frac{pa}{(a+b)(a+c)}$ | 0 | *p*/*a* | A | M |
| 7 | Sim | Simpson | $s_{Sim}=\frac{a}{\min\{(a+b),(a+c)\}}$ | 0 | 1 | A | N |
| 8 | BB | Braun-Blanquet | $s_{BB}=\frac{a}{\max\{(a+b),(a+c)\}}$ | 0 | 1 | A | M |
| 9 | DK | Driver-Kroeber, Ochiai, cosine | $s_{DK}=\frac{a}{\sqrt{(a+b)(a+c)}}$ | 0 | 1 | A | N |
| 10 | BUB | Baroni-Urbani-Buser | $s_{BUB}=\frac{\sqrt{ad}+a}{\sqrt{ad}+a+b+c}$ | 0 | 1 | I | M |
| 11 | Kul | Kulczynski | $s_{Kul}=\frac{1}{2}\left(\frac{a}{a+b}+\frac{a}{a+c}\right)$ | 0 | 1 | A | N |
| 12 | SS1 | Sokal-Sneath (1) | $s_{SS1}=\frac{a}{a+2b+2c}$ | 0 | 1 | A | M |
| 13 | SS2 | Sokal-Sneath (2) | $s_{SS2}=\frac{2a+2d}{p+a+d}$ | 0 | 1 | S | N |
| 14 | Ja | Jaccard | $s_{Ja}=\frac{3a}{3a+b+c}$ | 0 | 1 | A | N |
| 15 | Fai | Faith | $s_{Fai}=\frac{a+0.5d}{p}$ | 0 | 1 | I | M |
| 16 | Mou | Mountford | $s_{Mou}=\frac{2a}{ab+ac+2bc}$ | 0 | 2 | A | M |
| 17 | Mic | Michael | $s_{Mic}=\frac{4(ad-bc)}{(a+d)^{2}+(b+c)^{2}}$ | 1 | 2 | Q | N |
| 18 | RG | Rogot-Goldberg | $s_{RG}=\frac{a}{2a+b+c}+\frac{d}{2d+b+c}$ | 0 | 1 | S | M |
| 19 | HD | Hawkins-Dotson | $s_{HD}=\frac{1}{2}\left(\frac{a}{a+b+c}+\frac{d}{b+c+d}\right)$ | 0 | 1 | S | M |
| 20 | Yu1 | Yule (1) | $s_{Yu1}=\frac{ad-bc}{ad+bc}$ | 1 | 2 | Q | N |
| 21 | Yu2 | Yule (2) | $s_{Yu2}=\frac{\sqrt{ad}-\sqrt{bc}}{\sqrt{ad}+\sqrt{bc}}$ | 1 | 2 | Q | M |
| 22 | Fos | Fossum | $s_{Fos}=\frac{p(a-0.5)^{2}}{(a+b)(a+c)}$ | 1 | (*p* – 0.5^2^)/*p* | A | M |
| 23 | Den | Dennis | $s_{Den}=\frac{ad-bc}{\sqrt{p(a+b)(a+c)}}$ | (*p*/2)^1/2^ | *p*^1/2^ | Q | M |
| 24 | Co1 | Cole (1) | $s_{Co1}=\frac{ad-bc}{(a+c)(c+d)}$ | *p* - 1 | *p* | Q | N |
| 25 | Co2 | Cole (2) | $s_{Co2}=\frac{ad-bc}{(a+b)(b+d)}$ | *p* - 1 | *p* | Q | N |
| 26 | dis | Dispersion | $s_{dis}=\frac{ad-bc}{p^{2}}$ | 1/4 | 1/2 | Q | N |
| 27 | GK | Goodman-Kruskal | $s_{GK}=\frac{2\min(a,d)-b-c}{2\min(a,d)+b+c}$ | 1 | 2 | S | N |
| 28 | SS3 | Sokal-Sneath (3) | $s_{SS3}=\frac{1}{4}\left(\frac{a}{a+b}+\frac{a}{a+c}+\frac{d}{b+d}+\frac{d}{c+d}\right)$ | 0 | 1 | S | M |
| 29 | SS4 | Sokal-Sneath (4) | $s_{SS4}=\frac{ad}{\sqrt{(a+b)(a+c)(b+d)(c+d)}}$ | 0 | 1 | S | M |
| 30 | Phi | Pearson-Heron colligation coefficient | $s_{Phi}=\frac{ad-bc}{\sqrt{(a+b)(a+c)(c+d)(b+d)}}$ | 1 | 2 | Q | M |
| 31 | Di1 | Dice (1) | $s_{Di1}=\frac{a}{a+b}$ | 0 | 1 | A | N |
| 32 | Di2 | Dice (2) | $s_{Di2}=\frac{a}{a+c}$ | 0 | 1 | A | N |
| 33 | Sor | Sorgenfrei | $s_{Sor}=\frac{a^{2}}{(a+b)(a+c)}$ | 0 | 1 | A | N |
| 34 | Coh | Cohen | $s_{Coh}=\frac{2(ad-bc)}{(a+b)(b+d)+(a+c)(c+d)}$ | 1 | 2 | Q | N |
| 35 | Pe1 | Peirce (1) | $s_{Pe1}=\frac{ad-bc}{(a+b)(c+d)}$ | 1 | 2 | Q | N |
| 36 | Pe2 | Peirce (2) | $s_{Pe2}=\frac{ad-bc}{(a+c)(b+d)}$ | 1 | 2 | Q | N |
| 37 | MP | Maxwell-Pilliner | $s_{MP}=\frac{2(ad-bc)}{(a+b)(c+d)+(a+c)(b+d)}$ | 1 | 2 | Q | M |
| 38 | HL | Harris-Lahey | $s_{HL}=\frac{a(2d+b+c)}{2(a+b+c)}+\frac{d(2a+b+c)}{2(b+c+d)}$ | 0 | *p* | S | N |
| 39 | CT1 | Consoni-Todeschini (1) | $s_{CT1}=\frac{\ln(1+a+d)}{\ln(1+p)}$ | 0 | 1 | S | M |
| 40 | CT2 | Consoni-Todeschini (2) | $s_{CT2}=\frac{\ln(1+p)-\ln(1+b+c)}{\ln(1+p)}$ | 0 | 1 | S | N |
| 41 | CT3 | Consoni-Todeschini (3) | $s_{CT3}=\frac{\ln(1+a)}{\ln(1+p)}$ | 0 | 1 | A | N |
| 42 | CT4 | Consoni-Todeschini (4) | $s_{CT4}=\frac{\ln(1+a)}{\ln(1+a+b+c)}$ | 0 | 1 | A | N |
| 43 | CT5 | Consoni-Todeschini (5) | $s_{CT5}=\frac{\ln(1+ad)-\ln(1+bc)}{\ln(1+p^{2}/4)}$ | 0 | 1 | S | M |
| 44 | AC | Austin-Colwell | $s_{AC}=\frac{2}{\pi}\arcsin\sqrt{\frac{a+d}{p}}$ | 0 | 1 | S | M |
